# Excluding nonuniversity-certified professionals from Spain’s personal training sector: a narrative review of epidemiological, legal, and economic implications

**DOI:** 10.1016/j.hpopen.2026.100186

**Published:** 2026-10-07

**Authors:** Gonzalo Quesada-Fernández, Santiago Jacomet Carrasco, Mario Muñoz-López, Marcos Gutiérrez-García, José Francisco López-Gil

**Affiliations:** a Faculty of Sport and Health Sciences, Fit Generation Research Institute, AD500 Andorra la Vella, Andorra; b Associação para a Promoção da Educação e Formação de Adultos (PROEFA), Coimbra, Portugal; c Orthos Formación, Barcelona, Spain; d School of Medicine, Universidad Espíritu Santo, Samborondón, Ecuador; e Vicerrectoría de Investigación y Postgrado, Universidad de Los Lagos, Osorno, Chile

**Keywords:** Public health policy, workforce regulation, injury prevention, fitness sector, health equity

## Abstract

**Background:**

Spain is considering restricting personal training to university graduates, which would exclude vocationally trained professionals (NUCPs).

**Objectives:**

To conduct an exploratory policy scenario analysis, integrating narrative evidence synthesis, descriptive epidemiology, legal proportionality assessment, and economic modeling, to examine potential public health, legal, and economic implications of excluding NUCPs, under the explicit assumption that such exclusion reduces client access to professional supervision.

**Methods:**

We synthesized legal, institutional, and scientific sources (January–May 2025) and developed illustrative scenario models using official workforce statistics and published injury and cost parameters.

**Results:**

Under a hypothetical supervision-reduction scenario, not empirically observed consequences of the regulation, illustrative projections suggested 8812 additional injury events annually (probabilistic 95% uncertainty interval: 3552–14,802) among clients losing supervision, with an estimated productivity cost from temporary work disability of €16,504,725 (direct healthcare costs not included). A supply elasticity model projected short-term price increases of 18% (Es = 0.5) to 30% (Es = 0.3). Available evidence did not demonstrate that university qualification per se reduces injury risk.

**Conclusions:**

The proposed exclusion lacks direct empirical support; modeled scenarios indicate potential adverse consequences under conservative assumptions. Less restrictive alternatives merit consideration.

## Highlights

• Excluding vocationally trained fitness professionals may reduce access to supervised exercise.
• Loss of supervision could be associated with ∼8900 additional injuries annually in Spain.
• Estimated direct healthcare costs may reach €16.7 million per year under this scenario.
• Workforce restrictions could increase service prices by ∼18%, affecting lower-income groups.
• Current evidence lacks qualification-specific data, limiting justification for exclusionary regulation.

## 1 Introduction

The personal training sector has experienced remarkable growth over recent decades, largely driven by increasing public awareness of the health benefits associated with regular physical activity [1], [2]. In Spain, however, this expansion has been accompanied by persistent debates regarding the regulation of professional qualifications and access to the sector [3]. The current regulatory framework remains fragmented, with substantial differences among autonomous communities and multiple educational pathways into professional practice, including university degrees, vocational programs, and certified training courses.

A particularly contentious issue currently under discussion concerns the proposed exclusion of Higher Technicians in Physical Conditioning (HTPC) and Certified Sports Technicians (CST) [4], collectively referred to in this paper as non-university-certified professionals (NUCP), in favor of graduates in Sports Science [4]. The *Colegio Oficial de Licenciados en Educación Física* (COLEF) has advocated for a new legal framework that would reserve key professional domains, such as health-oriented exercise, physical rehabilitation, performance optimization, and social inclusion, exclusively for university-trained professionals [5]. If enacted, this measure would effectively disqualify NUCP from practicing in these areas, raising serious concerns regarding its implications for user safety, service accessibility, labor market stability, and the overall economic sustainability of the sector.

This study does not seek to establish a direct causal relationship between trainers' academic background and user safety, as such data are not yet available within the Spanish context. Instead, it explores whether the exclusion of vocationally trained professionals could plausibly generate negative outcomes, such as increased injury risk, based on existing epidemiological evidence and supervision contexts.

From a legal standpoint, the study applies the proportionality test as defined by the Court of Justice of the European Union (CJEU) [6]. This three-stage test assesses whether a restriction is (1) suitable for achieving its intended aim, (2) necessary in the absence of less restrictive alternatives, and (3) proportionate in the strict sense, ensuring that the potential benefits outweigh its broader social and economic costs. The draft bill currently under review justifies the proposed exclusion by citing concerns related to user safety and professional standards [7], claiming that up to 50% of current sector workers lack adequate formal training. The proposed regulation would therefore mandate specific qualifications, create a national registry of accredited trainers, introduce sanctions for non-accredited practice, and provide a transitional period for currently active NUCPs.

At the European level, however, no directive requires a university degree to practice as a personal trainer. In contrast, reports from the European Commission and its Expert Group on Skills and Human Resource Development in Sport emphasize the value of competence-based qualification systems, which recognize vocational and certified learning as legitimate routes into the profession [8]. The PEAK Framework [9], promoted by the European Commission, similarly supports a pluralistic model that prioritizes demonstrable professional competence over academic credentials.

In this context, the present study pursues three main objectives: (1) to examine whether available evidence supports the claim that excluding NUCPs would enhance safety or reduce adverse outcomes in personal training; (2) to estimate the potential impact of such exclusion on workforce availability, service pricing, and accessibility; and (3) to evaluate whether the proposed regulation satisfies the proportionality test or whether less restrictive alternatives could achieve the same objectives without undermining safety or equity.

## 2 Materials and methods

This study is an exploratory policy scenario analysis based on narrative evidence synthesis. It integrates: (1) structured review of legal, institutional, and epidemiological sources; (2) descriptive analysis of secondary workforce and injury data; and (3) transparent scenario modeling to illustrate plausible consequences if NUCP exclusion reduces supervision access. It does not estimate causal effects of academic qualification on injury risk, for which no disaggregated data exist in Spain.

This narrative policy impact assessment adopts a multidisciplinary, policy-oriented approach to evaluate the implications of excluding non-university-certified professionals (NUCP) from key professional domains within Spain's personal training sector. The analysis integrates two complementary dimensions:

1. A descriptive epidemiological and socioeconomic evaluation of the current structure of personal training services in Spain, and
2. A proportionality assessment of the proposed regulatory measure, following the jurisprudence of the CJEU.

Rather than relying on experimental or cohort-based designs, this study synthesizes secondary evidence from epidemiological reports, legislative documents, official statistics, economic analyses, and technical consensus statements [10]. These sources were selected to provide an integrated perspective on injury rates, service accessibility, professional qualifications, and labor market dynamics.

The first dimension compiles and organizes quantitative indicators from national and regional reports [11], allowing the identification of patterns in sports-related injuries and the estimation of potential impacts derived from restricting professional access. These data underpin the empirical assessment of whether the purported benefits of excluding NUCP, particularly regarding safety and quality, are plausible.

The second dimension applies the principle of proportionality, a legal standard used to assess the legitimacy of regulatory restrictions on fundamental rights [12]. This three-step test examines:

1) Suitability (i.e., whether the measure can achieve its intended objective).
2) Necessity (i.e., whether that objective could be achieved through less restrictive means).
3) Proportionality in the strict sense (i.e., whether the benefits outweigh the broader social, economic, and accessibility costs).

By combining empirical evidence and structured legal reasoning, this approach seeks to determine whether the proposed exclusion of NUCP is justified in terms of public safety and professional competence, or whether it represents an excessive and unnecessary barrier to professional practice [13]. All data were analyzed within the scope of their original sources, and no primary data collection or experimental manipulation was undertaken.

### 2.1 Search strategy

The search strategy was implemented between January and May 2025, integrating legal, institutional, and scientific evidence. The process began with a review of official Spanish gazettes, the *Boletín Oficial del Estado* [14] and the *Boletín de las Cortes Generales* [15], focusing on Law 6/2016 of the Community of Madrid [16] and the Sports Law 39/2022 [17]. At the European level, reference frameworks included the European Skills, Competences, Qualifications and Occupations (ESCO) classification [18], the Qualifications Directive [19], and the Proportionality Test Directive [20].

### 2.2 Review type and reporting

This evidence synthesis was conducted as a narrative review to inform an exploratory policy scenario analysis. It was not designed, conducted, or reported as a systematic review; therefore, PRISMA guidelines do not fully apply. To maximize transparency, we report search sources, date range, search terms, selection criteria, and the distinction between the core analytic corpus and supporting references (Supplementary Material S1). Source quality was appraised for relevance, institutional authority, and direct applicability rather than through formal risk-of-bias tools.

Complementary national data were obtained from ministerial reports (Labour, Health, and Treasury) and official statistical sources, particularly from the *Instituto Nacional de Estadística* (INE) [21] and the Spanish Social Security System [22].

A parallel search was conducted in PubMed, Scopus, and Web of Science using combinations of the keywords “personal trainer”, “university degree”, “vocational training”, “safety”, “injuries”, “quality”, and “regulation”.

The multidisciplinary search design allowed the integration of legal and institutional sources with scientific literature to assess the impact of excluding NUCP on safety, quality, and accessibility. No studies were found directly evaluating whether the proposed restriction meets the proportionality principle or whether less restrictive measures could achieve the same policy goals.

### 2.3 Data collection and processing

Data collection was based on a structured review of secondary documents published by institutional, academic, and regulatory bodies [23]. The final core analytic corpus consisted of six documents selected for full structured extraction because of their direct relevance to the relationship between professional qualification, safety, accessibility, and service quality in personal training in Spain.

Included documents comprised national and regional epidemiological reports, studies on injuries in recreational and professional settings, sectoral analyses of the Spanish sports ecosystem, and publications addressing legislative or regulatory developments.

Inclusion criteria were as follows:

1. Addressed the relationship between physical activity and safety outcomes (injuries, accidents, hospitalizations, or mortality).
2. Provided data specific to Spain or comparable EU countries.
3. Contained quantitative estimates of incidence, prevalence, or socioeconomic consequences of sports-related injuries.
4. Were published by official institutions, peer-reviewed journals, or recognized professional organizations.

Data extraction was conducted systematically using a Guided Qualitative Framework (GQF), which organized information by source, publication year, population, training context, and key outcomes. More than 40 additional references were used as supporting sources for context, official statistics, methodology, or legal background and did not undergo full GQF extraction (Supplementary Table S1). This enabled cross-comparison of regulated versus unregulated environments and identification of major data gaps, especially the absence of disaggregated data by trainer qualification.

As none of the reviewed sources provided causal evidence linking trainer certification with injury or mortality, the analysis focused on describing general trends, comparing regulatory contexts, and estimating plausible indirect effects of professional exclusion on safety and accessibility [24]. This approach situates the study's hypotheses within an empirically grounded yet policy-relevant framework.

### 2.4 Statistical analysis

Given the heterogeneity of data sources and the absence of primary data, the statistical analysis employed a descriptive, modeling-based approach to estimate the epidemiological and economic implications of excluding NUCP [25].

The analysis proceeded in two stages:

1. Descriptive assessment: Epidemiological indicators such as injury incidence and prevalence per 1000 participants were extracted from published sources, stratified (when possible) by supervision status, sport type, and training setting. Relative risks were used to approximate differences between supervised and unsupervised environments and to infer the potential protective role of professional oversight [26].
2. Hypothetical burden modeling: A projection model was developed to estimate the additional injuries likely to result from reduced trainer availability, combining national participation data with injury rates from supervised and unsupervised contexts. The estimated injury differential was monetized using published Spanish estimates of the cost of temporary work disability attributable to musculoskeletal disease (Lázaro et al. [27], which do not include direct healthcare expenditure.

Multi-parameter sensitivity analyses (vocational share, trainer:client ratio, reference prevalence, RR, unit cost, and supply elasticity) were conducted in R. Results are reported in Supplementary Tables S2-S6 and Fig. S1-S3. No missing-data imputation was performed on source datasets. Model-derived estimates, including the supervised injury proportion (reference prevalence × RR), were calculated arithmetically from published parameters. Calculations retained full numerical precision throughout; counts and monetary estimates were rounded only for presentation. Probabilistic sensitivity analysis used 10,000 Monte Carlo simulations with independent triangular distributions for the vocational workforce share (minimum/mode/maximum: 0.05/0.091/0.15), clients per trainer (10/20/30), reference injury prevalence (0.20/0.42/0.473), supervised risk ratio (RR) (0.60/0.67/0.75), and cost per temporary-work-disability episode (€1200/€1873/€2500). The number of registered workers was held fixed at 34,933. Uncertainty intervals were defined by the 2.5^th^ and 97.5^th^ percentiles of the simulated outputs.

### 2.5 Price impact estimation through supply elasticity modeling

To quantify potential market effects, a supply elasticity model was used to estimate short-term changes in personal training service prices following a reduction in workforce availability.

The model assumes:

%ΔP=%ΔQ/Eₛ

where:

• %ΔP is the estimated change in price,
• %ΔQ is the percentage change in supply, and
• Eₛ is the short-run elasticity of supply.

Following Industrial Economics, Inc. (2023) [28], Eₛ was set at 0.5, consistent with elasticity estimates for credential-constrained personal services (e.g., healthcare, childcare). This provides a conservative basis for the fitness sector, where similar certification barriers apply. Additionally, a linear regression analysis was conducted to assess temporal trends in student enrollment between 2019 and 2024 across two educational pathways: HTPC and university degrees. For each pathway, enrollment counts were modeled as a function of academic year, with an interaction term to test for differential trends. A two-tailed significance level of *p* < 0.05 was applied. Model assumptions were verified through graphical and formal diagnostics to confirm compliance with Gauss–Markov conditions (linearity, independence, homoscedasticity, and absence of multicollinearity). All analyses were conducted using RStudio (Posit Software, PBC, Boston, MA, 2024).

### 2.6 Ethical considerations

This study did not involve human participants, experiments, or primary data collection. All information was obtained from publicly accessible sources, including government publications, institutional datasets, and peer-reviewed literature. No personal or sensitive data were used. In accordance with international standards for secondary data research, ethical approval was not required [29].

## 3 Results

### 3.1 Descriptive epidemiology of sports injuries in Spain

Epidemiological data obtained from institutional reports and peer-reviewed literature indicate a high burden of sports-related injuries across various population groups in Spain. The most relevant indicators are summarized below.

In primary care settings, between 10% and 20% of consultations for musculoskeletal complaints are attributable to physical activity or sports injuries. The most frequently associated activities include football, basketball, and cycling, followed by running, swimming, and gym-based exercise. The most commonly reported diagnoses are sprains, contusions, tendinopathies, and lumbar syndromes.

Among urban amateur runners, a nationwide epidemiological survey reported a 12-month injury prevalence of 42%.

According to data from the Instituto Nacional de Salud (INS), more than one million sports-related injuries are treated annually in Spain. In supervised physical activity programs targeting weight loss, the prevalence of musculoskeletal injuries is estimated at 47.3% [30]. Unsupervised and non-individualized training programs, especially those involving exercise beyond an individual's technical ability, can lead to severe injuries or even death, highlighting the importance of professional guidance and proper planning [31]. Among runners, the overall injury rate was 20.8 injuries per 100 female runners and 20.4 injuries per 100 male runners. In a nationwide survey of individuals injured during recreational sports in Spain, football accounted for the highest proportion of injuries (27.6%), followed by running (8.6%), indoor football (7.9%), and basketball (7.7%), out of a total of 1616 reported cases [32].

In 2018, the Spanish sports industry generated economic activity of €39.1 billion, representing 3.3% of the national GDP, and supported nearly 414,000 jobs, equivalent to 2.1% of the employed population [33] (Table 1).

**Table 1:** Key epidemiological indicators from national reports and studies in Spain.

Indicator	Value/range	Population/context	Source
Total injuries from recreational sports (12 months); most injury-causing sports	1616 injuries. Most frequent: football (27.6%), running (8.6%), indoor football (7.9%), basketball (7.7%)	Injured people surveyed in Spain	García González [32]
Prevalence of musculoskeletal injuries in supervised weight-loss exercise programs	47.3%	Adults with overweight/obesity in 12-week programs	Butragueño-Revenga [30]
Injury incidence in amateur runners	3.2–84.9%	10,941 runners	AMF–SEMFYC [31]
Proportion of injured runners	20.6 per 100 runners	10,941 runners (Women/men mean)	AMF–SEMFYC
Economic contribution of the sports industry	€39.1 billion (3.3% of GDP); 413,966 jobs (2.1% of total employment)	Spanish general population	PwC report based on INE input–output tables (2018) [33]

Table 2 shows the average annual expenditure on sports-related goods and services per person in 2023, stratified by educational attainment [34]. Individuals with higher education spent an average of €193 per year, nearly 2.5 times more than those with only primary or lower secondary education (€80.4). This gradient reflects underlying socioeconomic inequalities in access to paid sports opportunities. The average for the total population was €131.8.

**Table 2:** Average annual sports-related expenditures per person by education level (2023).

Educational level	Average annual expenditure (€)
Primary or lower secondary education	80.4
Upper secondary education	110.0
Higher education or equivalent	193.0
**Total population (all levels)**	131.8

### 3.2 Effectiveness of supervised versus unsupervised injury prevention programs

A recent meta-analysis by Valentin et al. [35], which included 44 randomized controlled trials and a total of 40,409 participants, assessed the effectiveness of exercise-based injury prevention programs (IPPs) in reducing musculoskeletal injuries in sports. The findings confirmed a statistically significant overall benefit of these programs, with a 29% reduction in injury risk compared with standard training (RR = 0.71; 95% confidence interval [CI]: 0.64–0.78; *p* < 0.001). However, when analyzed by supervision status, clear differences in effectiveness emerged.

Supervised IPPs were associated with a 33% reduction in injury risk (RR = 0.67; 95% CI: 0.60–0.75; *p* < 0.001). In contrast, in the unsupervised programs, supervision did not have a statistically significant effect (RR = 1.04; 95% CI: 0.90–1.19; *p* = 0.580).

The lack of efficacy observed in unsupervised interventions may be partly explained by lower adherence rates and suboptimal execution of exercise, both of which are difficult to ensure in the absence of in-person supervision. The meta-regression analysis revealed a significant inverse association between adherence and injury risk (β = −0.014; p = 0.004), suggesting that the more consistently participants adhered to the program, the greater the reduction in injury rates (Table 3).

**Table 3:** Key Findings from Valentin et al. (2023).

Variable	Finding	RR	*p* value
Overall program effect	Injury risk reduced by 29%	0.71 [0.64–0.78]	< 0.001
Supervised IPPs	Injury risk reduced by 33%	0.67 [0.60–0.75]	< 0.001
Unsupervised IPPs	No significant effect	1.04 [0.90–1.19]	0.580

**Note**: *IPPs*, injury prevention programs. *RR*, risk ratio.

### 3.3 Distribution of registered workers in gym activities

According to official data from the Spanish Social Security system (May 2025), 34,933 individuals are formally registered under economic activity code 93.13, corresponding to “gymnasium activities” [36]. Of these, the vast majority are employed under the general social security scheme, whereas a substantially smaller fraction is registered as self-employed. The number of self-employed workers in this sector is notably low, totalling 7214 individuals.

Between the academic years of 2019–2020 and 2023–2024, enrollment in both HTPC programs and university degrees in sport-related fields increased steadily. HTPC programs grew from 4687 to 10,530 students, whereas university degree enrollment rose from 20,984 to 23,875 students (Fig. 1). Linear regression analysis confirmed these upwards trends. HTPC programs showed a statistically significant yearly increase of 1459 students (95% CI: 960–1957; *p* = 0.003), and university degrees increased by 730 students per year (95% CI: 369–1091; *p* = 0.008). The interaction model revealed that the rate of growth was significantly greater for the HTPC pathway than for the university pathway (β = 729, *p* = 0.009).

**Fig. 1:**
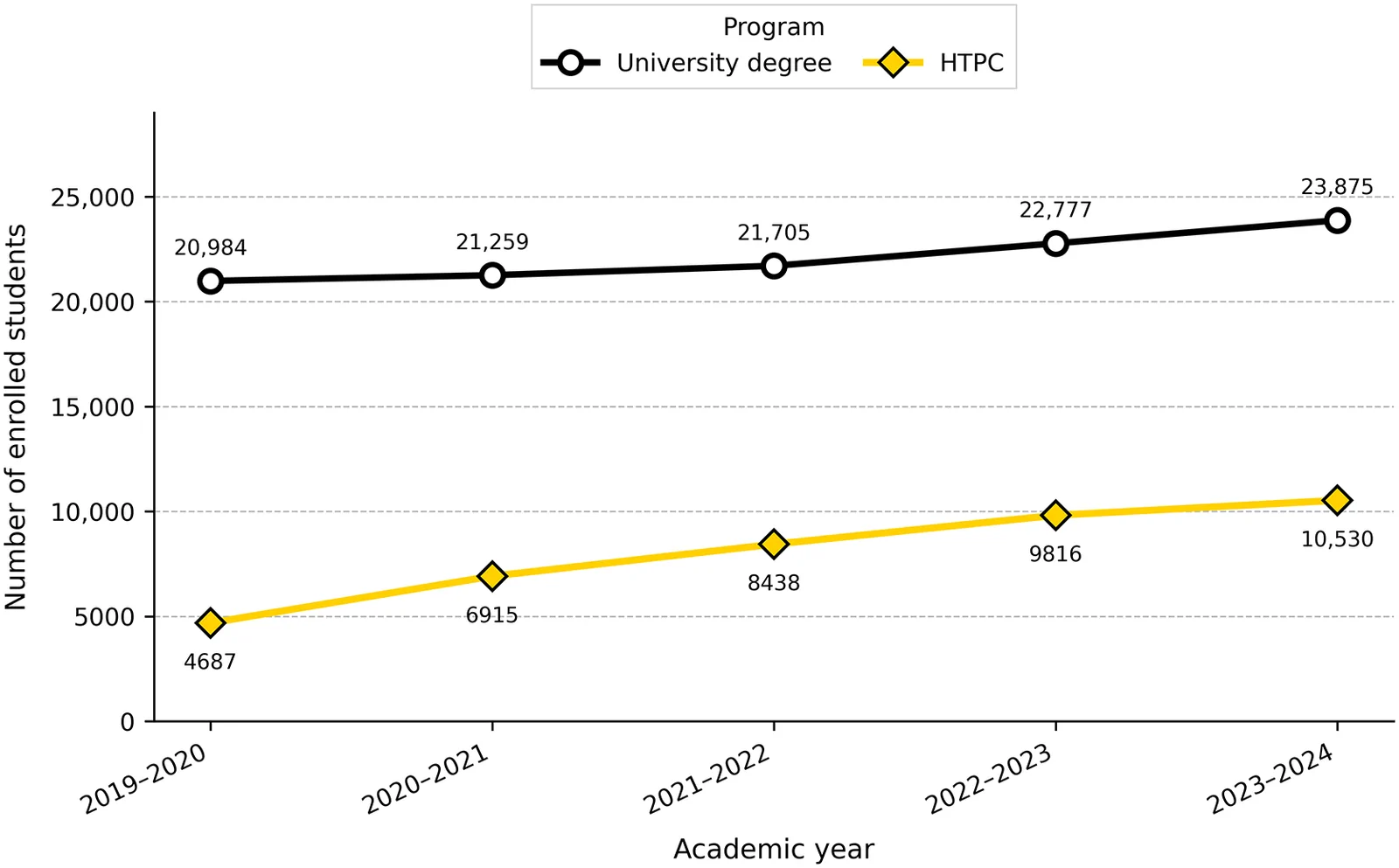
Trends in student enrollment in university and Higher Technicians in Physical Conditioning programs in Spain (2019–2024). Source: Anuario de Estadísticas Deportivas 2025, Table 5.1. Ministry of Education, Vocational Training and Sports.

### 3.4 Workforce composition in the Spanish sport sector

According to the 2024 statistical summary published by the Ministry of Culture and Sport [37] (Table 4), the sport-related workforce in Spain comprises approximately 256,000 individuals, representing 1.2% of the total national employment. Among them, 51.4% (n ≈ 131,600) hold higher education qualifications, 29.6% (n ≈ 75,800) have completed upper secondary education, and 9.1% (n ≈ 23,300) hold vocational training credentials, including programs such as NUCP.

**Table 4:** Average annual employment linked to sports-related occupations or activities in Spain, by sex, age group, and educational level (2021–2024).

	ABSOLUTE VALUES (in thousands)				As % of total employment		Employment distribution		% of total employment	
	2021	2022	2023	2024	2021	2022	2023	2024	2023	2024
**TOTAL**	222.9	234.6	246.7	256.0	1.1	1.1	1.2	1.2	100.0	100.0
**SEX**										
Men	124.6	135.6	148.3	152.6	1.2	1.2	1.3	1.3	60.1	59.8
Women	98.4	98.9	98.4	102.4	1.1	1.0	1.0	1.0	39.9	40.2

**AGE GROUP**										
16–24 years	37.8	52.5	67.8	72.3	3.8	4.6	5.6	5.5	27.5	28.3
25–34 years	67.0	61.1	66.3	64.3	1.8	1.6	1.6	1.6	26.9	25.2
35–44 years	49.7	51.3	47.0	45.5	0.9	0.9	0.9	0.8	19.1	17.8
45–54 years	49.1	46.1	38.0	44.3	0.9	0.8	0.6	0.7	15.4	17.4
≥55 years	19.3	23.5	27.5	28.7	0.5	0.6	0.6	0.6	11.2	11.2

**EDUCATIONAL LEVEL**										
Lower secondary or less	36.5	40.9	41.1	48.7	0.6	0.7	0.6	0.8	16.7	19.1
Upper secondary education	64.9	73.3	82.0	75.4	1.4	1.5	1.6	1.5	33.2	29.6
— General track	46.6	48.2	57.3	52.2	1.7	1.7	2.0	1.8	23.2	20.5
— Vocational track	18.3	25.1	24.7	23.2	0.9	1.2	1.1	1.0	10.0	9.1
Higher education or equivalent	121.5	120.4	123.6	131.0	1.3	1.3	1.3	1.3	50.1	51.4

**Source:** DEPORTEData. Ministry of Education, Vocational Training and Sports. The National Statistics Institute (INE) is based on the Labour Force Survey (EPA). Annual averages. Subgroup absolute values may not sum exactly to total because of rounding of annual averages.

Part-time employment accounts for 43.5% of the total sport workforce. Self-employment is reported in 14.8% of cases. These figures refer to the entire sector and do not provide specific data on the number of personal trainers or their distribution by qualification.

### 3.5 Estimated injury risk by supervision status

Critical interpretive distinction. The scenario model quantifies potential consequences of reduced professional supervision, drawing on meta-analytic evidence that supervised injury-prevention programs reduce injury risk relative to unsupervised contexts (RR = 0.67). This evidence does not establish that university-qualified trainers deliver safer supervision than NUCPs. No reviewed source provided injury data disaggregated by academic credential. Projected figures represent illustrative scenarios under stated assumptions, not empirically demonstrated regulatory impacts.

To estimate the potential impact of excluding NUCP from the personal training sector, this study uses two benchmark values for injury incidence: one for supervised exercise contexts and one for unsupervised contexts. These values are derived from epidemiological sources described in the previous sections and serve as inputs for the subsequent burden model.

For the unsupervised scenario, the 12-month injury prevalence was set at 42%, on the basis of a nationwide survey of urban amateur runners in Spain. This population, which typically engages in self-directed training without real-time professional guidance, reported a 12-month injury prevalence of 42.1%. Additional national data support this figure, indicating that the majority of sports-related injuries in Spain occur in informal or unsupervised contexts.

For the supervised scenario, the injury incidence was estimated by applying the effect size reported in a meta-analysis by Valentin et al. This study revealed that compared with standard or unsupervised training, supervised injury prevention programs reduced the risk of musculoskeletal injuries by 33% (RR = 0.67; 95% CI: 0.60–0.75; *p* < 0.001).

Using the 42% unsupervised injury rate as the reference, the corresponding supervised injury rate was estimated by multiplying this baseline by the RR reported in the meta-analysis:

Supervised injury incidence=42%×0.67=28.14%

This yields a model-derived injury proportion of approximately 28% under supervised conditions. This approach allows the model to incorporate a realistic, empirically grounded estimate of the protective effect of professional supervision rather than relying on raw incidence values from highly specific or high-risk populations. The reported prevalence of 42.1% was rounded to 42.0% for the baseline scenario.

### 3.6 Estimated population affected by the regulatory change

The sequential model proceeds as follows: (1) 34,933 CNAE 93.13 workers [observed]; (2) × 9.1% vocational share [sector extrapolation]; (3) = 3179 displaced NUCPs [modeled]; (4) × 1:20 trainer:client ratio [assumption, sensitivity 1:10–1:30]; (5) = 63,578 affected clients [modeled]; (6) model-derived injury proportions 28.1% (supervised) vs 42.0% (unsupervised reference prevalence) [derived: 42% × RR 0.67]; (7) = 8812 excess events [model output]; (8) × €1873 temporary-work-disability unit cost = €16.5 M [model output; productivity losses only, excluding direct healthcare]. Parameter sensitivity ranges are reported in Supplementary Table S2.

According to the most recent data from the Spanish Social Security system (May 2025) [36], a total of 34,933 individuals are formally registered under economic activity code 93.13, corresponding to “gymnasium activities”. Of these, 27,719 are employed under the general social security scheme, whereas 7214 are registered as self-employed.

Although official statistics do not disaggregate this group by professional qualifications, workforce data from the Ministry of Culture and Sport (2025) [37] indicate that 9.1% of individuals employed in the broader sport sector hold vocational training credentials, including the NUCP.

Applying this proportion directly to the gym sector yields an estimate of 3179 vocationally trained professionals (9.1% of 34,933). However, this is likely a conservative underestimate, since vocational qualifications are disproportionately represented in gym-based employment relative to other sport contexts (e.g., federated coaching, management, or education) [38]. In the absence of official disaggregated data and to avoid overestimation, this conservative baseline is retained for modeling purposes.

To estimate the number of gym users who would potentially lose access to professional supervision following the exclusion of NUCP, a supervision ratio of 1 trainer per 20 clients was applied. This figure reflects the upper limit of supervision ratios recommended by international professional bodies, such as the National Strength and Conditioning Association (NSCA), and aligns with macrolevel modeling standards. Although actual trainer-to-client ratios are often lower in individual or small-group formats, the 1:20 ratio serves as a cautious and standardized reference.

Multiplying the estimated 3179 displaced professionals by 20 yields an affected population of 63,578 individuals who may lose access to structured supervision in gym environments under the modeled scenario. This figure is used in subsequent sections to project changes in injury burden and estimate the corresponding productivity-related economic burden associated with reduced professional coverage.

### 3.7 Differential injury rates by supervision status

To estimate the health impact of reduced professional supervision in gym environments, model-derived injury proportions for supervised and unsupervised exercise contexts were applied to the affected population previously estimated.

As established in the preceding sections, the reference 12-month injury prevalence for unsupervised contexts is set at 42%, on the basis of epidemiological data from urban amateur runners in Spain. For supervised contexts, a model-derived injury proportion of 28% was calculated by applying the 33% RR reduction reported in a meta-analysis of supervised injury prevention programs [35].

These values are applied to the estimated 63,578 individuals who would potentially lose access to professional supervision under the modeled scenario: With professional supervision: expected injuries = 63,578.06 × 0.2814 ≈ 17,891 per year. Without professional supervision: expected injuries = 63,578.06 × 0.42 ≈ 26,703 per year.

This differential yields 8812 modeled excess injury events annually under the assumed supervision-reduction scenario. These figures are scenario outputs conditional on reduced access to professional supervision; they are not empirically demonstrated consequences of an enacted regulation, and they do not estimate effects of academic qualification per se.

This estimate serves as the basis for calculating the attributable risk and projecting the potential economic burden derived from the increase in injury burden (Fig. S2).

### 3.8 Sensitivity range on the basis of confidence intervals

The RR of injury with supervised exercise versus unsupervised exercise, as reported in the meta-analysis (RR = 0.67; 95% CI: 0.60–0.75), allows for an estimation of uncertainty around the projected excess injury cases. Applying the lower and upper bounds of the CI yields supervised injury rates of 25.2% and 31.5%, respectively. This results in a projected range of modeled excess injuries under the assumed supervision-reduction scenario of 6676 to 10,681 cases per year.

The range of 6676–10,681 excess injuries reflects uncertainty in the RR alone, with all other parameters held fixed. The separate Monte Carlo analysis produced a 95% uncertainty interval of 3552–14,802 excess injuries per year (Supplementary Table S5 and Fig. S3).

### 3.9 Estimated economic burden attributable to loss of professional supervision

The unit cost of €1873 is taken from Lázaro et al. [27] and represents the mean cost per episode of temporary work disability (incapacidad temporal) attributable to musculoskeletal disease in Spain, based on 2007 Social Security data. This estimate reflects sick-leave-related (indirect/productivity) costs borne by the National Institute of Social Security, mutual insurers, and employers. It does not include direct healthcare expenditure (primary care, diagnostics, or treatment). Accordingly, the €16,504,725 estimate represents illustrative productivity losses under the modeled scenario, not healthcare expenditure. Direct medical costs were not estimated and would be additional. Not all modeled injury events would generate a temporary-work-disability episode of average duration (approximately 43 days). The 2007 parameter should be interpreted as a directional, dated input rather than a current healthcare-system cost.

On the basis of the baseline estimate of 8812 modeled excess injuries per year under the assumed supervision-reduction scenario, the estimated productivity cost from temporary work disability was calculated using published unit costs. According to official data, the mean cost per musculoskeletal injury episode in Spain, attributable to temporary work disability and excluding direct healthcare expenditure, is €1873 [27] (2007 data).

Using this unit cost to the projected number of excess injuries yields the following estimate:

Estimatedannualproductivitycost:34,933×0.091×20×0.42×(1–0.67)×€1873≈€16,504,725(Excessinjuryeventsroundedforpresentation:8812)

This figure represents the estimated annual productivity cost from temporary work disability only (€16,504,725) (Supplementary Table S6). It should not be interpreted as healthcare-system expenditure. Direct medical costs were not estimated and would be additional; not all modeled injury events would generate a temporary-work-disability episode of average duration.

Holding the unit cost at €1873 and varying only the RR across its 95% confidence interval yields an estimated annual productivity cost of approximately €12.5–20.0 million.

### 3.10 Price impact estimation through supply elasticity modeling

To estimate the potential effect of excluding NUCP on service affordability, a supply elasticity model was applied (Supplementary Table S4).

According to official data from the Spanish Ministry of Culture and Sport (2025) [37], approximately 9.1% of the total sport-related workforce holds vocational training credentials, including the NUCP. Assuming that this same proportion applies to the 34,933 professionals currently working in gym-related activities (*Clasificación Nacional de Actividades Económicas* [CNAE] 93.13), the exclusion of NUCP would result in an estimated 9.1% contraction in supply.

On the basis of sectoral analyses by Industrial Economics, Inc. (2023) [28], short-run price elasticities of supply for licensed or credential-constrained service professions typically range between 0.3 and 0.8. For the personal training sector, where qualification requirements limit workforce flexibility, an elasticity of 0.5 is considered a conservative and realistic assumption.

The elasticity formula is as follows:

%ΔP=%ΔQ/Eₛ

$$\%\mathrm{\Delta P}=\left(9.1\%\right)/0.5=18.2\%$$

Under these assumptions, the exclusion of vocationally trained professionals would be expected to cause a short-term increase of approximately 18% in personal training service prices.

### 3.11 Application of the proportionality test: quantitative assessment

The proportionality of the proposed exclusion was evaluated in accordance with the European legal standard, which comprises three cumulative elements (Table 5):

1. *Suitability.* There is no empirical evidence demonstrating that academic qualifications are causally superior to vocational training in reducing injury risk. Both types of qualifications are formally recognized within national and European regulatory frameworks.
2. Necessity. Exclusion affects 3179 NUCPs currently employed in gym-related activities under the modelled baseline. Assuming a supervision ratio of 1 trainer per 20 clients [39], an estimated 63,578 users would lose access to supervised training. On the basis of model-derived injury proportions (28.1% under supervision versus 42.0% without supervision), the baseline scenario yields 8812 modeled excess injuries per year. The attributable risk increase (ARI) used in the continuous model is 0.1386.
3. Proportionality in the strict sense. The estimated productivity burden associated with modeled excess injuries is €16,504,725 annually, based on a unit cost of €1873 per temporary-work-disability episode (sick leave only; excluding healthcare). In parallel, the exclusion would reduce the supply of trainers by 9.1%, and given a conservative supply elasticity of 0.5, this would result in an estimated 18.2% increase in service prices.

**Table 5:** Application of the proportionality test to exclude NUCP in Spain.

Criterion	Description	Assessment
Suitability	The measure must be suitable to achieve its objective (e.g., improved safety and quality).	Not demonstrated. No evidence indicates superior safety outcomes among university graduates compared to NUCP.
Necessity	The measure must be the least restrictive option available to achieve the objective.	Not fulfilled. Less restrictive alternatives (certification exams, continuing education, accreditation) are available.
Proportionality (Strict Sense)	The benefits of the measure must outweigh the negative impact on other rights and interests.	Not justified. Disproportionate effects on employment, access, equity, and public health without clear gain in safety.

## 4 Discussion

The results of this analysis raise substantial concerns about the scientific foundation, legal proportionality, and public health implications of the proposed exclusion of vocationally trained professionals, specifically those holding non-university credentials, from the personal training sector in Spain. While the regulation purports to enhance safety and service quality by limiting practice to university graduates, the evidence presented in this study does not support the notion that such a measure would produce the intended outcomes. In contrast, excluding qualified vocational professionals would likely reduce access to supervision, increase injury burden, exacerbate health inequities, and generate substantial productivity losses from temporary work disability (€16,504,725 in the baseline scenario). Direct healthcare expenditure was not included in this monetization and remains an additional, unestimated cost domain.

This exclusion also ignores structural changes in the educational landscape. Between the academic years of 2019–2020 and 2023–2024, enrollment in both HTPC programs and university degrees in sport-related fields increased steadily. HTPC programs more than doubled, rising from 4687 to 10,530 students, whereas university degree enrollment increased from 20,984 to 23,875. Linear regression analysis confirmed these trends: HTPC programs showed a statistically significant yearly increase of 1459 students (95% CI: 960–1957; *p* = 0.003), and university degrees increased by 730 students per year (95% CI: 369–1091; *p* = 0.008). The interaction model revealed that the rate of growth was significantly greater for HTPC than for university pathways (β = 729, *p* = 0.009). These findings, illustrated in Fig. 1, highlight the rapid expansion and growing relevance of non-university professional qualifications in the fitness sector.

From an epidemiological standpoint and on the basis of the available indirect evidence, the difference in injury incidence between supervised and unsupervised environments is substantial. Drawing on validated Spanish surveillance data and international meta-analyses, the annual injury risk increases from 28.1% under professional supervision to 42.0% without professional supervision. When applied to the 63,578 users who would potentially lose supervised access under the assumed exclusion scenario, this translates to 8812 modeled excess injuries annually. While the relative contrast is sizable, these figures remain illustrative projections under stated assumptions rather than observed effects of the proposed regulation.

The associated economic implications are substantive under the modeled scenario. Using published unit cost estimates for musculoskeletal injuries in Spain, the illustrative projection yields an annual productivity burden from temporary work disability of €16,504,725. This figure excludes direct healthcare expenditure and longer-term effects such as disability, comorbidities, or psychosocial harm, and should be regarded as a conservative directional estimate. Taken together, these scenario projections suggest that the proposed measure could introduce a substantial productivity burden from sick leave, with additional unestimated healthcare costs, and with no offsetting demonstrable safety benefit, placing the onus on regulatory proponents to provide evidence that NUCPs deliver inferior client safety outcomes.

Importantly, current epidemiological records do not disaggregate injury data according to the specific type of professional qualification. As such, causal claims comparing university versus vocational credentials cannot be directly substantiated. However, the available evidence strongly supports the protective role of supervision per se, regardless of academic background, suggesting that a broader inclusion of qualified professionals may better serve public health and equity objectives.

Furthermore, the results suggest that the proposed regulatory change would distort the market for personal training services. On the basis of sector-specific elasticity data from regulated professions [28], the short-run price elasticity of supply for credential-constrained services such as personal training can be conservatively estimated at 0.5. Under this assumption and considering a supply reduction of approximately 9.1% following the exclusion of NUCP, the model projects a short-term price increase of 18% to 30%, depending on the elasticity scenario. While these figures are lower than previously anticipated, they remain significant, especially for price-sensitive populations in low-income or underserved areas, where alternative supervised options may be unavailable. These findings run counter to public health efforts aimed at expanding equitable access to physical activity for prevention and rehabilitation.

Importantly, the assumption that university graduates inherently provide safer or higher-quality services than vocationally trained professionals lack an empirical foundation. Epidemiological studies do not report injury incidence disaggregated by academic credential, and no evidence has been presented by the Spanish authorities or the COLEF establishing a causal link between university-level education and improved client outcomes.

A scoping review by Linton et al. [40] identified supervision and structured support as central pillars of injury prevention strategies in runners. In their synthesis of 31 randomized controlled trials, interventions that included supervision, whether in person, through digital platforms, or via scheduled feedback, consistently outperformed unsupervised alternatives. Strength training and gait retraining were particularly effective under professional guidance, whereas isolated or generic interventions such as online modules or footwear recommendations showed limited efficacy.

Crucially, the benefits of supervision identified in the review were not restricted to NUCP. In contrast, coaches and vocationally trained personnel play a central role in injury prevention, participant adherence, and risk perception.

These findings suggest that the value of supervision in sport and exercise contexts does not hinge exclusively on academic qualifications. Legislative initiatives that seek to restrict supervision roles to graduates in physical activity and sport sciences may overlook that injury-prevention benefits documented in the literature are linked to supervised and structured support rather than to university credentials per se. This study does not demonstrate that NUCP qualification itself reduces injury risk; rather, it indicates that policies reducing access to professional supervision could increase modeled injury burden if displaced vocational capacity is not replaced.

### 4.1 Legal discussions

The European policy framework further undermines the legitimacy of the exclusionary measure. Both the European Qualifications Framework (EQF) and sector-specific instruments such as the PEAK framework recognize multiple valid pathways into professional practice, emphasizing competence over academic origin. The restriction proposed in Spain deviates sharply from this pluralistic model, introducing a form of educational exclusivity that appears both unnecessary and misaligned with EU principles of proportionality and non-discrimination.

The proportionality assessment, conducted according to the tripartite test established by the CJEU, further reinforces these conclusions. First, the appropriateness of the measure has not been demonstrated, as no evidence has been provided that it would achieve the objective of increased safety. Second, its necessity is questionable given the availability of less restrictive alternatives, such as certification exams, continuous training requirements, or oversight mechanisms, that could achieve the same goal without excluding an entire segment of the workforce. Third, in the strict sense of proportionality, the measure fails to balance its hypothetical benefits against its demonstrated costs.

A comparative perspective with past decisions by the Court of Justice of the European Union (CJEU) and the Spanish Constitutional Court (TC) reinforces the legal critique of the proposed exclusion. In CJEU Case C-55/94 (Gebhard), the Court established that any restriction on the freedom to provide services must be justified by imperative reasons in the general interest, be suitable for securing the attainment of the objective, and not exceed what is necessary. Considering this precedent, the exclusion of NUCP appears to be neither necessary nor proportionate in achieving improved safety or quality standards in personal training services.

Finally, from a public health ethics perspective, the policy may inadvertently exacerbate the very risks it aims to reduce.

In sum, the exclusion of NUCP from personal training roles in Spain is not supported by direct empirical evidence on scientific, economic, or legal grounds. The results presented in this study provide no direct empirical evidence that such a measure would produce significant harm without measurable benefit, raising serious questions about its legitimacy, its proportionality, and its alignment with European norms. These concerns warrant close scrutiny by regulators, legal scholars, and public health authorities alike.

### 4.2 Critique of the Colegio Oficial de Licenciados en Educación Física (COLEF) proportionality test framework

Considering the preceding legal analysis, critically assessing the main document used to support the proposed exclusion is essential: *“La profesión de la Educación Física y Deportiva y su regulación”* (Consejo COLEF, 2023) [41]. Although the report aims to justify the restriction of professional activities to university graduates in physical activity and sport sciences by means of a proportionality test, it fails to meet the standards established under Directive (EU) 2018/958 and its transposition into Spanish law.

Despite exceeding 700 pages, the document lacks a structured application of the proportionality test. It provides extensive background on the history of the profession, academic curricula, and labor market, but these sections offer little to no direct evidence supporting the restriction. The document appears to build its case by accumulation rather than demonstration, relying on tangentially related arguments rather than empirical data.

The report has several critical weaknesses:

1. *Overstated concept of public interest*. While the benefits of physical activity are well documented, they cannot be attributed solely to university-qualified professionals. No data are provided to demonstrate a concrete public health risk associated with the NUCP.
2. *Lack of causal evidence*. The report fails to establish a cause–effect relationship between the trainer's academic background and user safety. Presenting parallel arguments does not substitute for causal analysis.
3. *Disregard for less restrictive alternatives*. The document does not adequately consider certification systems, continuing education, or recognition of experience, which are commonly accepted mechanisms within the European Qualifications Framework.
4. *Exaggeration of necessity*. The proposed scope of restricted activities is overly broad. One section claims that there are “no alternative pathways to acquire the same qualification”, a claim that is asserted but not substantiated.
5. *Weak international comparisons*. The report selectively references international regulations and misrepresents instruments such as the ESCO framework, which in fact supports competency-based access to professions, contrary to the conclusions of the report.
6. *Disproportionate measures*. The proposed regulation fails to align the level of risk involved in personal training activities with the level of qualification needed, violating the principle of proportionality.

This misrepresentation of the evidence can also be seen in how the COLEF report cites academic literature to support its safety claims. For example, the report cites Huxley, O'Connor, and Healey [42] to support the claim that inadequate trainer qualifications are a documented cause of injuries in youth athletes. However, this interpretation misrepresents the original study. The article is a retrospective analysis focused on the Australian coaching system, and it does not establish any causal or statistical relationship between trainer credentials and injury rates. Rather, it highlights a gap in specific training for coaches of young athletes, noting that only 4.8% had completed a professional development course titled “Coaching the Young Athlete”. Importantly, this recommended qualification is not equivalent to a university degree but rather to a lower specialized Level 4 coaching certificate. Therefore, the source does not support the assertion that university-level qualifications are necessary to ensure safety, nor does it demonstrate that vocational qualifications are inherently inadequate.

A similar pattern of misinterpretation is found in the use of Bianchi et al. [43], which the COLEF report cites as supporting their claim, despite the study showing no such evidence. The COLEF report cites this research as evidence that training under NUCP increases injury risk. However, the authors conducted a retrospective cross-sectional analysis and reported a non-significant difference in injury incidence between clients trained by university graduates (5.5 injuries per 1000 person-months; 95% CI: 3.1–9.2) and those trained by individuals with alternative qualifications (6.3; 95% CI: 4.2–9.2). The incidence rate ratio (IRR) was 1.1 (95% CI: 0.6–2.3; *p* = 0.348), indicating that there was no statistically significant association. Given the cross-sectional design, no causal inference can be drawn. Moreover, potential confounders, such as socioeconomic status influencing trainer selection and healthcare access, were not controlled for.

In summary, the COLEF document does not fulfill the minimum legal or evidentiary standards required for a well-founded proportionality justification under Directive (EU) 2018/958.

### 4.3 Overview of Spain's fitness coach training system and its implications

Spain's fitness non-university training system is characterized by a strong institutional framework that combines scientific, methodological, and practical competencies [38]. Curricula often include core subjects such as anatomy, physiology, and exercise psychology, alongside modules focused on communication, ethics, and professional conduct. This integrative approach ensures that fitness professionals are not only technically competent but also socially and ethically prepared to interact with diverse clientele.

Importantly, the emphasis on supervised practice and the requirement of formal certification contribute to a professional environment where trainers are accountable to recognized standards. These features align well with public health goals, as they help mitigate risks associated with unsupervised or inadequately trained practitioners.

The implications are twofold: first, attempts to exclude NUCP must consider the strength and outcomes of existing vocational pathways; second, the current system appears well positioned to support both injury prevention and professional accountability, especially when complemented by robust oversight.

### 4.4 Methodological considerations

Our study has several important limitations. First, the analysis of injury incidence and safety outcomes is based on descriptive epidemiological data. None of the included sources disaggregate injury or mortality data according to the academic qualifications of the professionals involved. The absence of official qualification-specific data introduces uncertainty and limits the ability to establish causal inferences regarding the relationship between trainer credentials and user safety. Second, the estimates concerning workforce exclusion and economic impact rely on secondary data sources and extrapolations from labor force surveys and institutional registries. Third, the proportionality analysis, although it is based on established European jurisprudence and legal doctrine, does not constitute a formal legal opinion or judicial interpretation. Fourth, the study does not include primary data collection or experimental analysis. Finally, this study should be understood as an exploratory policy analysis based on indirect evidence. Further studies employing longitudinal designs, injury surveillance systems, and data stratified by professional qualifications are essential to address the public health and regulatory questions raised by the proposed policy.

## 5 Conclusions

This exploratory scenario analysis suggests that excluding NUCPs from Spain's personal training sector may have adverse implications for service accessibility and, under the explicit assumption that such exclusion reduces client access to professional supervision, for modeled injury burden and productivity-related costs (€16,504,725 in the baseline scenario)—without clear empirical justification that university qualification per se improves safety. Projected effects depend on that supervision-access assumption and on parameters described in the Methods and Supplement; they should not be interpreted as demonstrated causal effects of the proposed regulation. While framed as a quality-enhancing measure, the proposed regulation does not appear proportionate to its stated objectives on the available evidence. Policymakers should consider less restrictive alternatives that uphold professional standards while preserving equity, workforce diversity, and public health outcomes.

## CRediT authorship contribution statement

**Gonzalo Quesada-Fernández:** Writing – original draft, Investigation, Formal analysis, Conceptualization. **Santiago Jacomet Carrasco:** Writing – review & editing. **Mario Muñoz-López:** Writing – review & editing. **Marcos Gutiérrez-García:** Writing – review & editing. **José Francisco López-Gil:** Writing – review & editing, Supervision.

## Consent for publication

Not applicable.

## Ethics approval and consent to participate

Not applicable.

## Funding

This research received no external funding.

## Declaration of competing interest

Three authors (GQ, MML, MGG) are affiliated with Fit Generation, a fitness education organization in Andorra that provides both university-level education, including a university degree in Physical Activity and Sport Sciences, and non-university fitness training, and whose institutional interests may therefore be directly affected by the proposed regulation. One author (SJC) is affiliated with *Orthos Formación*, a vocational sports training school in Barcelona, Spain, whose institutional interests are similarly affected by the regulatory outcome examined in this study. These affiliations constitute potential institutional conflicts of interest that readers should bear in mind when interpreting the findings. The remaining author (JFL-G) declares no competing interests. No author received financial compensation related to this study, and no external funder or sponsor had any role in study design, data collection, analysis, interpretation, or writing.

## Data Availability

All inputs are publicly available official or published sources cited in the manuscript. The tabulated outputs are provided as supplementary material.
