# Supplementary material for: Excluding nonuniversity-certified professionals from Spain’s personal training sector: a narrative review of epidemiological, legal, and economic implications
Source: Health Policy Open. 2026 Oct 7;11:100186. doi: 10.1016/j.hpopen.2026.100186 (PMC13694889; doi:10.1016/j.hpopen.2026.100186)
Supplement: Supplementary file 1 [file mmc1.docx]

# Supplementary material

# Supplementary Material S1. Narrative review search and selection.

## Review design

This supplementary material describes the search and selection procedures for the narrative evidence synthesis underpinning the policy scenario analysis. The review was purposive and multidisciplinary, integrating scientific literature, legal and institutional documents, and official statistics. It was not a systematic review and is therefore not reported in accordance with PRISMA 2020.

## Search objectives

Searches aimed to identify evidence on: (1) professional qualification, supervision, and injury or safety outcomes in exercise settings; (2) workforce structure and regulation of personal training in Spain and comparable EU contexts; (3) legal proportionality frameworks for professional restrictions; and (4) economic parameters for scenario modelling.

## Sources searched

Scientific databases: PubMed, Scopus, and Web of Science (January-May 2025).
Grey and institutional sources: Boletin Oficial del Estado, Boletin de las Cortes Generales, Ministry of Education, Culture and Sport reports, Spanish Social Security statistics, INE, EU official publications (ESCO, Qualifications Directive, Proportionality Test Directive), and documents from professional bodies (including COLEF).

## Search strings (scientific databases)

PubMed: ("personal trainer" OR "fitness professional" OR "exercise professional") AND (qualification OR certification OR regulation OR safety OR injur*).
Scopus/Web of Science: equivalent combinations using TITLE-ABS-KEY fields, without language restrictions where applicable.

## Selection process

Records were screened iteratively by title and abstract for direct relevance to the study questions. Full texts were retrieved when potentially eligible. Selection was conducted by one lead reviewer and checked by a second reviewer; disagreements were resolved by discussion. The process continued until informational saturation was reached for each analytic dimension (qualification/safety, workforce, injury burden, economics, and legal proportionality).

## Inclusion criteria

(1) Addressed physical activity and safety outcomes, professional qualification, regulation, or service accessibility; (2) provided data for Spain or comparable EU countries where possible; (3) contained quantitative or regulatory information usable in the policy analysis; (4) were published by peer-reviewed journals, official institutions, or recognised professional bodies.

## Exclusion criteria

Records were excluded if they lacked direct relevance to personal training regulation, did not contribute usable evidence to any analytic dimension, or duplicated information already captured in a higher-quality source.

## Core analytic corpus versus supporting references

Six documents underwent full structured extraction using the Guided Qualitative Framework (GQF) because they directly informed the qualification-safety-accessibility triad. More than 40 additional references were cited for contextual background, official statistics, methodological support, or legal precedents; these were not subjected to full GQF extraction. Supplementary Table S1 maps sources to each analytic dimension.

## Source appraisal

Formal risk-of-bias tools were not applied because sources were heterogeneous (policy, legal, epidemiological, and economic). Instead, sources were appraised for relevance, timeliness, institutional authority, and direct applicability to the Spanish policy question.

# Supplementary Table S1. Source mapping by analytic dimension.

| **Dimension** | **Core sources (GQF)** | **Supporting references** |
| --- | --- | --- |
| Qualification vs safety | Bianchi et al.; Valentin et al.; COLEF report | Legal frameworks; EU directives |
| Workforce composition | Social Security CNAE 93.13; Ministry Culture and Sport 2024 | Boned et al. fitness club profile |
| Injury burden | Runner prevalence survey; INEFC epidemiology; Butragueño-Revenga | INS national estimates |
| Economic costs | Lazaro et al. 2014 (temporary work disability / sick leave, 2007) | PwC sports industry report |
| Legal proportionality | CJEU jurisprudence; Directive 2018/958; STC precedents | Van Gestel and Van Lochem |

## Supplementary Table S2. Scenario sensitivity matrix.

| Scenario | Vocational share (%) | Trainer:client ratio | Reference prevalence (%) | RR supervised | TWD cost per episode (EUR) | NUCPs (n) | Clients (n) | Excess injuries (n) | Total cost (EUR) |
| --- | --- | --- | --- | --- | --- | --- | --- | --- | --- |
| Optimistic | 5.0 | 10 | 20.0 | 0.75 | 1200 | 1747 | 17,466 | 873 | 1,047,990 |
| Baseline | 9.1 | 20 | 42.0 | 0.67 | 1873 | 3179 | 63,578 | 8812 | 16,504,725 |
| Pessimistic | 15.0 | 30 | 47.3 | 0.60 | 2500 | 5240 | 15,7198 | 29,742 | 74,354,890 |

## Supplementary Table S3. One-way sensitivity analysis.

| **Parameter** | **Low** | **Base** | **High** | **Excess injuries (low)** | **Excess injuries (base)** | **Excess injuries (high)** | **Range (n)** |
| --- | --- | --- | --- | --- | --- | --- | --- |
| Vocational share (9.1% sector applied to gym workforce) | 5.0 | 9.1 | 15.0 | 4842 | 8812 | 14525 | 9683 |
| Trainer:client ratio | 10 | 20 | 30 | 4406 | 8812 | 13218 | 8812 |
| 12-mo injury prevalence (reference pop.) | 20.0 | 42.0 | 47.3 | 4196 | 8812 | 9924 | 5728 |
| RR supervised vs unsupervised (Valentin) | 0.60 | 0.67 | 0.75 | 10,681 | 8812 | 6676 | 4005 |
| CNAE 93.13 registered workers | 33,000 | 34,933 | 37,000 | 8324 | 8812 | 9333 | 1009 |
| Cost per TWD episode (EUR; sick leave only) | 1200 | 1873 | 2500 | 8812 | 8812 | 8812 | 0 |

## Supplementary Table S4. Price elasticity scenarios

| **Supply elasticity (Es)** | **Supply reduction (%)** | **Price increase (%)** |
| --- | --- | --- |
| 0.3 | 9.1 | 30.3 |
| 0.4 | 9.1 | 22.7 |
| 0.5 | 9.1 | 18.2 |
| 0.6 | 9.1 | 15.2 |
| 0.8 | 9.1 | 11.4 |

## Supplementary Table S5. Probabilistic uncertainty (10,000 Monte Carlo draws).

| **Outcome** | **2.5^th^ percentile** | **Median** | **97.5^th^ percentile** |
| --- | --- | --- | --- |
| Excess injuries (n/year) | 3552 | 7666 | 14,802 |
| Total cost (EUR/year) | 6,228,969 | 14,086,859 | 28,587,312 |

## Supplementary Table S6. Summary of key model outputs.

| **Item** | **Value** |
| --- | --- |
| Baseline excess injuries | 8812 |
| Baseline total cost (EUR) | 16,504,725 |
| RR-only CI lower (injuries) | 6676 |
| RR-only CI upper (injuries) | 10,681 |
| Optimistic scenario injuries | 873 |
| Pessimistic scenario injuries | 29,742 |
| MC median injuries | 7666 |
| MC 95% UI injuries | 3552-14,802 |
| Price increase E=0.5 (%) | 18.2% |
| Price increase E=0.3 (%) | 30.3% |

## Supplementary Figure S1. One-way sensitivity (tornado plot).

## Supplementary Figure S2. Two-way sensitivity: vocational share and trainer-client ratio.

## Supplementary Figure S3. Probabilistic distribution of excess injuries.

## Supplementary Figure S4. Student enrolment trends (2019-2024).
